# Supplementary material for: Inhaled Placental Mesenchymal Stromal Cell Secretome from Two- and Three-Dimensional Cell Cultures Promotes Survival and Regeneration in Acute Lung Injury Model in Mice
Source: Int J Mol Sci. 2022 Mar 22;23(7):3417. doi: 10.3390/ijms23073417 (PMC8998959; doi:10.3390/ijms23073417)
Supplement: Supplementary file 1 [file ijms-23-03417-s001.zip › ijms-1571006-supplementary.pdf]

International Journal of *Molecular Sciences*

Supplementary Materials

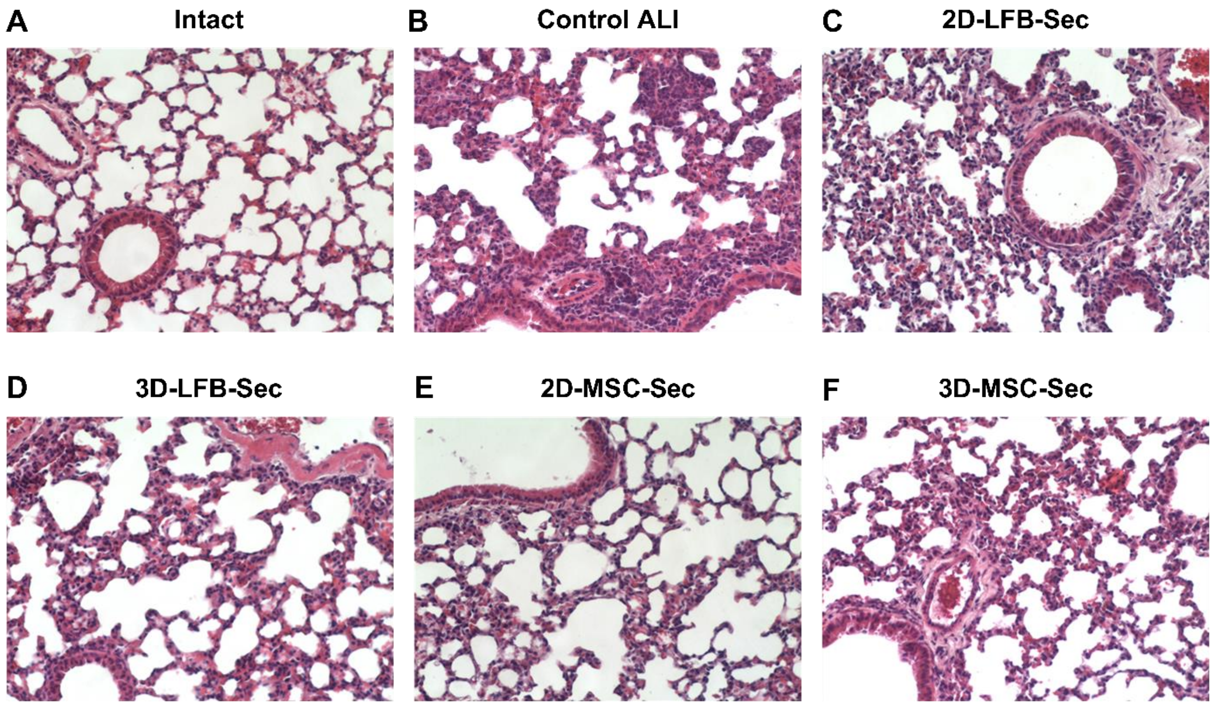

Figure S1. Histology examination of the lungs. (**A**–**F**) Lung tissue structure in the control group and on day 8 of LPS-induced ALI, either in nontreated mice or after lyophilizate inhalation. Sections were stained with hematoxylin and eosin; ×200 magnification. LPS, lipopolysaccharide; ALI, acute lung injury; MSC, mesenchymal stromal cell; LFB, lung fibroblast.
